# Can Housing Assets Affect the Chinese Residents’ Willingness to Pay for Green Housing?

**DOI:** 10.3389/fpsyg.2021.782035

**Published:** 2022-01-24

**Authors:** Qian Wu, Ziyang Zheng, Wenbo Li

**Affiliations:** ^1^Business School, Jiangsu Normal University, Xuzhou, China; ^2^Department of Real Estate, Konkuk University, Seoul, South Korea

**Keywords:** green housing, individual happiness, housing assets, ivprobit model, willingness to pay

## Abstract

As the development trend of the future housing field, green housing is an effective way to reduce pollution, save energy, and promote industrial upgrading. At the same time, the green house is of great significance to change the development mode of the construction industry and promote the sustainable development of the social economy. This study proposes a comprehensive research model to examine the influencing mechanism of residents’ intention to purchase green buildings. The proposed model is empirically tested using data collected from 1,338 urban residents in China. Based on logit, probit, and ivprobit models, factors such as personal characteristics, housing price, and the number of real estate ownership are selected to conduct empirical analysis and mechanism analysis on willingness that affects consumers’ purchase of green houses. The results show that housing assets significantly affect the willingness of householders to pay for green houses. The more houses they own, the higher their willingness to pay for a green house will be. Similarly, if the housing prices are higher, householders are more willing to buy a green house. The amount of housing assets will affect the willingness of householders to pay for green housing through the way of individual happiness. In terms of the characteristics of the householder, if the householder is more educated, unmarried, his willingness to buy a green house will be stronger, and owning housing assets may affect the individual happiness due to the housing wealth effect brought by rising housing prices. People with more housing assets are more likely to have the happiness brought by higher wealth, which may affect the purchase intention of householders.

## Introduction

The traditional construction industry is characterized by high energy consumption and low efficiency, and it is in urgent need of transformation and upgrading, so, green housing came into being (CABEE, 2017). As a new type of housing model, green housing emphasizes the importance of saving resources and protecting the environment, conforms to the concept of sustainable development, and is also a new trend in the development of the real estate and construction industry in the future (Darko and Chan, 2016). Green housing contains strong development potential and market opportunities in the Chinese real estate market. The development of green buildings is not only a policy requirement of China but also a long-term plan based on the future life of the people across the nation.

However, green housing has the characteristics of low popularity and high prices compared with ordinary housing. People’s concerns about the high purchase cost of green housing and the later investment income led to great uncertainty in consumers’ purchase intention of green housing. However, the purchase intention of consumers is affected by many factors and finally failed to form a green housing consumption market.

Following the increased awareness of its benefits, standards that define green housing began to develop worldwide. Compared with other developed countries, the green building development in China lags behind that of developed countries (Jones and Laquidara-Carr, 2016). More than 95% of buildings in China are high-energy-consuming buildings, and their energy consumption is about three times that of other developed countries. In recent years, under the guidance of relevant national policies, the development of green buildings in China has accelerated (Li, 2016; Ma et al., 2017).

China is a newly emerged green building market (Data, 2016) with only 5% of Chinese enterprises implementing green building projects, accounting for more than 60% of the total construction projects. One major reason for low green building consumption in China is the low residents’ demand intentions for green housing.

Especially from the year 2019, the housing sector has suffered numerous difficulties from the coronavirus disease 2019 (COVID-19) pandemic, including the field of green buildings, and this problem will last for a long time. During this special period, it has led to a great deal of uncertainty in many areas of residential life, and home became much more important for living even for working, thus, suitable environments must be enabled. That means COVID-19 is bound to cause new requirements for future housing which involves resource efficiency (Kaklauskas et al., 2021). Future green building assessment will likely focus more on its occupants than the building itself.

Therefore, it is necessary to explore consumers’ purchase intention of green housing in China, analyze and determine which factors directly or indirectly affect the purchase intention, and get the deepest influencing factors. Therefore, it is urgent to explore consumers’ willingness to buy green housing (Wang et al., 2015). This study will provide a helpful reference for government departments to formulate relevant policies and effective measurements to improve housing comfort, safety, and health; stimulate the green housing market; and achieve sustainable development of the Chinese real estate economy.

On the basis of research, this study further explores how to promote the popularity of green housing in the Chinese real estate market and increase people’s awareness of green constructions.

## Literature Review

This study conducts empirical research on green housing consumers from a micro perspective. After obtaining a large amount of real and accurate data through field research, we scientifically analyze the purchase intention of green housing consumers, and clarify how the purchase behavior of green housing arises and the influencing factors of purchase intention and the mechanism of multi-housing householders’ willingness to pay for green housing. Therefore, this article adopts a micro-quantitative research method to conduct in-depth and systematic research, and analyze effects of the main factors especially housing assets on Chinese residents willingness to pay for green housing, which can be as a reference for the promotion of green housing.

### Green Building Definition

In this article, the definition of green housing connotation refers to the definition of green building. The concept of green building originated from the word “ecological building,” which was proposed by The American architect Paolo Soleri in the 1960s. In 1990, the world’s first green building standard was released in the United Kingdom. Since then, green building has become the development direction of the industry, and various countries have launched their own green building evaluation standards, up to now, green housing has received increased attention over the past 20 years. In October 2000, the “International Conference on Sustainable Building 2000” was held in the Netherlands, which marked the comprehensive development of the international green building movement (Li et al., 2018). At the 20th UIA congress in Beijing in the year 1999, Architecture and Environment in the 21st Century were one of the important topics. The Beijing Charter published by UIA emphasized: “we must face the ecological dilemma to strengthen ecological awareness, at the same time, call upon architects all over the world to regard environmental and societal sustainable development as the core of their profession and responsibilities (Wu, 2000).”

The green building definition in China includes private and public green housing buildings (Assessment Standard for Green Building, 2016). Therefore, green housing is a subset of green buildings, with all the general characteristics of green buildings and some additional attributes (Li et al., 2018). Green housing emphasizes residence comfort, safety, and health in the context of livable space. It is an architectural concept to meet modern development requirements, but does not require a specific housing type, nor does it distinguish between regions. According to the Green Building Evaluation Standard of China, green housing can save resources (energy, land, water, and materials), protect the environment, and reduce pollution to the maximum extent, provide healthy, applicable, and efficient space for people, and harmoniously co-exist with nature within the whole life cycle of the building.

Since the COVID-19 pandemic arises, lockdowns in China have changed the way people and communities live, interact, and work, it also reminds us about the necessity to make the built environment resilient, including outdoor spaces, but especially indoor spaces, such as offices and entertainment facilities. Lockdowns tested the three main aspects of residential buildings, i.e., comfort, environment, and health and safety, which means new green housings should integrate wellbeing and hygiene (Kaklauskas et al., 2021).

### Research on the Influencing Factors of Green Housing Purchase Intention

Regarding the research on the influencing factors of green housing purchase intention, the Eastern and Western scholars have done some research, covering a wide range of angles. The main influencing factors are analyzed in the following categories, and finally combined with the influencing factors of residential purchase intention and green product purchase intention, it is combined to identify the research factors of green housing purchase behaviors.

#### Socio-Demographic Characteristics

The basic inquiry in examining the factors that affect the purchasing intention of the consumer is the relationship between socio-demographic factors and consumption. According to Jayantha and Ming (2016), the demographic background of the housing buyers, including income level, age, and marital status could influence the decision on property purchase. Zhang and Cai (2015) believed that the characteristics of residents have a significant impact on the purchase of green housing. It is found that the annual income level of the family determines the affordability of the buyers, which has a direct impact on the purchase intention when the price of a green house is higher than that of an ordinary house (Zhao and Chen, 2020). Besides, women may be more likely than men to be actively involved in improving the green environment (Belaid and Garcia, 2016). Furthermore, research reveals that the improvement in education has positive effects on low-carbon consumption behavior (Ding et al., 2017). Attaran and Celik (2015) investigated the environmental responsibility of students and their willingness to pay for studying and living in green buildings at New England University and found that female students are more environmentally responsible than male students.

#### Psychological Factors

According to Ding et al. (2018), the psychological factors include environmental value, personal norm, sense of responsibility, attitude, perceived behavioral control, subjective norms, intention, habits, and so on. The literature on green housing consumption emphasizes the influences of green attitudes, perceptions, and economic benefits on a consumer’s tendency to prefer green products that are on green purchase behavior (Rosner et al., 2021). Li et al. (2019) confirmed that the framing effect can influence the willingness of residents to pay for green housing through the resident environmental values. Egoistic and ecological values showed partial mediation between perceived benefits and willingness to pay for both framing contexts. Zhao and Chen (2021) found that perceived value is a crucial predictor of green housing purchase intention. Huang (2014) used the structural equation model to systematically study the willingness of consumers to pay for green housing, and through questionnaire surveys, it was found that factors such as consumer economy, social status, consumer expectations, consumer perception, external stimuli, and other factors have significant impacts on willingness to pay. Wang et al. (2015) analyzed the supply-demand relationship of China’s energy-saving building market and pointed out that factors, such as energy-saving building prices, preferences, future forecasts, energy-saving awareness of consumers, investment payback period, and corporate marketing and publicity have significant effects on the market demand for energy-saving buildings. Besides, personal norms are indirectly (through New Ecological Paradigm, in turn, awareness of consequences and ascription of responsibility) influenced by values, and they significantly have a direct effect on the green consumption behavior of residents (Fornara et al., 2016).

#### Economic and Political Factors

Policies are one of the most important external factors that affect the green consumption behavior of residents, and the policy instruments include information policy, economic policy, technology policy, and administrative regulation (Lindén et al., 2006).

Information policy mainly means information feedback. The economic policy mainly refers to “tax and subsidy” and “price.” The technical policy mainly affects the maturity of technology, and administrative regulations refer to mandatory policies that have a direct effect on residents’ green consumption behavior (Ding et al., 2018). Economic policy, as an external incentive, has a positive effect on environmental-friendly behavior and energy-saving behavior (Belaid and Garcia, 2016). Wang et al. (2015) studied the green housing market from a demand-side perspective and pointed out that consumers do not have a sufficient understanding of incremental costs, incremental benefits, and indirect incremental benefits, resulting in low purchase enthusiasm. The article analyzed the composition of green building incremental costs and established a green building market and finally suggested that government incentive subsidies should be considered to make indirect benefits explicit. Yang and Li (2014) studied the demand for the willingness of consumers to pay for green buildings and established a classification index of consumer demand factors for green buildings, starting from the three aspects of resource utilization, indoor environment, and community environment. Finally, they proposed that green building related knowledge affects the willingness of consumers to buy green housing. Zhang (2011) studied the willingness of consumers to pay for green buildings and analyzed factors, such as product feature stimulus, social stimulus, green advertising, and green certification, and found that the above factors have different effects on the willingness of consumers to pay and payment level. Robinson et al. (2016) analyzed the demand for green office building features among office tenants in the United States and determined that public firms and firms in the energy and information technology industries are most likely to pay for green-labeled buildings. Construction practitioners with a potential green housing purchasing intention are characteristic of informed consumers. Government economic incentives for green housing and the formulation of laws and regulations, evaluation standard, and release related to the concept of science popularization and propaganda can increase the perceived benefits of the green house, reduce the green housing’s perception of the cost, increase the trust and purchasing power of the consumer, and real estate enterprises in the promotion. The popularization of green housing related concept knowledge by the government can enhance the attention of residents to green housing and promote the formation of the green consumption concept of consumers in housing purchases (Zhao and Chen, 2020).

From the research results of the above scholars, it can be seen that in the research on the maturity of the green housing market, the main factors that affect purchase intentions of consumers are the speed and level of economic development, demographic characteristics, consumer product awareness, sales prices, environmental protection knowledge, social ethics, publicity, economic ability, and green housing product performance.

### The Impact of Housing Assets on Residential Consumption (Housing Wealth Effect)

This article aimed to investigate the key factors affecting the willingness of Chinese residents to pay for green housing for the following two reasons. First, basic sociodemographic characteristics, such as age, gender, occupation, income, and educational level are the influential factors identified from the above research. Second, to evaluate the willingness to pay for green housing by different socioeconomic groups in China and to reveal that rich and multi-housing householders prefer to pay more to improve their living comfort.

According to China Family Panel Studies (CFPS) survey, the impacts on urban Chinese consumers’ spending associated with housing value, financial assets, and household income are evaluated. Findings suggest that the housing assets play a significant effect on household consumption in China, which is much larger in comparison with developed countries. Scholars reveal that larger impact is related to structural limits on investing in all probability which favors real estate ownership, as well as the dominant position of housing in household wealth (Chen et al., 2021). Besides, Chinese residents who have joint ownership of housing property on average have the highest consumption propensity, while those having sole ownership of housing property consume the most in response to the appreciation in housing wealth (Chen et al., 2020).

Case et al. (2005) analyzed the macro panel data of 14 Western countries and the United States and found that changes in total housing wealth have a significant impact on total consumption, and its impact is much greater than that of financial wealth. A 10% increase in housing wealth will increase household consumption by 1 and 0.4% in Western countries and the United States, respectively. Carroll et al. (2017) distinguished between immediate and final wealth effects. When housing wealth increases, the recent marginal propensity to consume in 3 months is about 2%, and the propensity to spend additional wealth in a few years is about 9%. Overall, the literature supports the active housing wealth effect.

The housing wealth effect varies from family to family. Liao et al. (2014) pointed out that higher house prices mean higher hidden rents, which hinders the housing wealth effect of homeowners who use houses as housing. Their views on the housing wealth effect have several implications. The wealth effect of households with multiple houses should be stronger. This effect should be greater among homeowners with a shorter life expectancy and a weaker inheritance motivation. For owners who want to reduce the size of their families and plan to reduce the size of their houses, the impact will also be greater. In addition, the housing wealth effect may depend on preventive savings motives. Consumption growth may be positively correlated with the predictable part of housing price growth because higher home values reduce the need for precautionary savings (Hu et al., 2014, 2020; Carroll et al., 2017). Finally, Buiter (2008) pointed out that the appreciation of housing prices redistributes wealth from short-term housing families to long-term housing families. Therefore, tenants and owners may experience different housing wealth effects, and these effects may be offset overall.

If there is a wealth effect in the real estate market, what factor will affect it? Whether to own the ownership of the house and the number of houses held is an important factor affecting the wealth effect of the housing market. Buiter (2008) believes that the increase in housing prices will cause the redistribution of wealth from house renters to owners. That is, the wealth effect of house owners is greater than that of house renters. Sinai and Souleles (2005) pointed out that high house prices mean higher implied rents, which in turn results in a smaller wealth effect for single-house holders, while households with multiple houses have a larger wealth effect. Finally, higher the market value of the real estate will weaken the demand for precautionary savings, which will bring about an increase in consumption. That is the wealth effect of real estate and the reverse change of the precautionary savings motives of an individual (Peng et al., 2019). In fact, according to Pratt and Zeckhauser (1987), investors with different risk aversion coefficients will have different expected marginal utility from the same consumption combination. That is, the consumption decision of the family is closely related to the risk aversion coefficient. On the other hand, the real estate market is extremely risky. Therefore, the risk attitude of investors and the wealth effect of real estate must have a certain influence on the relationship.

Through combing the existing literature, there is no research that analyzes the relationship between the purchase intention of green housing and the wealth effect of housing assets from a family perspective. This article will investigate the willingness to pay of multi-housing householders for green housing using a large amount of questionnaire data, which can enrich the scope of sociodemographic characteristic groups in related literature. Additionally, it can offer valuable strategic recommendations for the government after the general consumers have gained sufficient knowledge about green housing. Therefore, the current study cannot only provide a theoretical basis for the guiding policies and operational strategies of the development of the green housing market but also provide an effective measure for the government to adjust the regulation and economic incentive policies related to upscaling the next step of green building promotion. So, the contributions of this study are idea innovation and data innovation.

## Data Sources and Research Methods

What are the deep reasons and the influencing paths of Chinese residents’ willingness to pay for green housing? This is the key question to promote green housing purchase behavior, and it is worth further study. Based on this, this article analyzes the relationship between housing wealth effects and green housing purchase intention, then explores the mechanism of multi-housing householders’ willingness to pay for green housing.

The survey sample covers 29 provinces (autonomous regions and municipalities), 1,338 households. The survey was conducted in 2019.

We take the willingness to pay for green housings of the respondents as an explained variable. According to the question in the survey, “Are you willing to pay for green housings?” the answers of “yes, I am willing to pay” are assigned a value of 1, answers of “no, I am not willing to pay” are assigned a value of 0.

The core explanatory variable is the number of house assets owned by the respondents (house number). In addition, the house price is one of the main variables discussed in this article. We choose the question “What is the market price of the house where your family currently lives” from the questionnaire as the data of house price (lnhouseprice).

In addition, considering other factors that may affect the willingness to pay for green housings of the respondents, we add a series of control variables in the empirical model based on previous literature, including demographic characteristics, household assets and income, and regional and urban characteristics. Demographic variables include gender (gender), education level (education), marriage (marriage), and family population (size). In the survey, the level of education is divided into nine categories. In this article, the value of vocational college and above is set as 1, otherwise, it is set as 0. In the category of household assets and income, total household income (lnincome) includes wage income, agricultural operating income, industrial and commercial operating income, transfer income, and investment income.

As for the regional characteristics, the urban households (rural) are assigned a value of “0,” and the rural households are assigned a value of “1” (see Table 1).

**TABLE 1:** Variable definition.

Variables	Definition
willingness	Will to pay for green housings takes 1, and not will to pay takes 0
housenumber	Number of housing assets owned by the householder
lnhouseprice	The market price of the house
gender	Male takes 1, female takes 0
education	College level and above is 1, otherwise is 0
marriage	Married or remarried take 1, otherwise take 0
size	Family population
lnincome	Total income in a family
rural	1 for living in rural areas, 0 for urban areas

## Model and Empirical Results

### Model Specification

Using the above data and variables, the logit and probit models are set to analyze the impact of housing on household risk attitudes. The basic model is as follows.


$$w\,i\,l\,l\,i\,n\,e\,s\,s_{i}=\alpha \,1\,h\,o\,u\,s\,e\,n\,u\,m\,b\,e\,r_{i}+\alpha \,2\,h\,o\,u\,s\,e\,p\,r\,i\,c\,e_{i}+\beta \,X_{i}+\varepsilon_{i}$$

In the model, willingness*_*i*_* represents the willingness to pay for green houses of the respondents *i*, house number represents the number of owner-occupied housing assets, and house price represents the market price of the house. *X*_*i*_ is the respondents or family characteristics, and ε_*i*_is the random error term.

### Descriptive Statistics

It can be seen from Table 2 that the education level of household heads is generally low. The average household size of the respondents was 3.58. The average gender is 0.5005, indicating that the proportion of male and female in the survey tends to be balanced. The average household income is ¥90,049.09.

**TABLE 2:** Descriptive statistics.

Variable	Obs	Mean	SD	Min	Max
willingness	1338	0.411357	0.49209	0	1
housenumber	1338	1.269564	0.590762	0	27
houseprice	1338	743721.7	1241674	2000	7.00E+06
gender	1338	0.500574	0.500003	0	1
education	1338	0.206829	0.405034	0	1
marriage	1338	0.022728	0.149035	0	1
size	1338	3.586014	1.52072	1	13
income	1338	90049.09	109884.3	−3460	695165
rural	1338	0.346083	0.475723	0	1

### Empirical Results

Table 3 reports the impact of house numbers and housing prices on the wiliness to pay for green houses of householders after using OLS, logit, and probit models. It can be found that after controlling other factors, if a family owns more housing assets, then the respondents will have a higher willingness to pay for green houses. Similarly, and if the housing price is higher, the respondents are more willing to take pay for green houses.

**TABLE 3:** Empirical results.

	Variables	OLS	Logit	Probit
willingness	housenumber	0.0451***	0.189***	0.117***
		−7.548	−7.31	−7.349
	lnhouseprice	0.0110***	0.0505***	0.0306***
		−4.99	−5.074	−5.068
	gender	−0.005	−0.0221	−0.0134
		(−0.757)	(−0.763)	(−0.755)
	education	0.135***	0.561***	0.350***
		−17.38	−16.9	−16.94
	marriage	−0.0552***	−0.262***	−0.158***
		(−3.588)	(−3.670)	(−3.682)
	size	0.0189***	0.0836***	0.0522***
		−8.556	−8.638	−8.765
	lnincome	0.00296	0.0129	0.00822
		−1.314	−1.308	−1.351
	rural	−0.0536***	−0.244***	−0.148***
		(−5.840)	(−5.913)	(−5.901)
		−1.691	(−10.99)	(−11.12)
	Constant	0.0651*	−1.895***	−1.171***
	Observations	1338	1338	1338

z-Statistics are in parentheses,***, **, and * indicate the significance levels of 1, 5, and 10%, respectively.

In addition, in the aspect of the characteristics of the household head, if the respondent has a higher education level, then he will have a stronger willingness to buy green houses. The married respondents prefer to buy green housing compared with those who are not married. In addition, compared with urban areas, households in rural areas are more inclined to buy green houses.

### Endogenous Solving

Considering that the above estimation results may be biased due to factors, such as reverse causality and missing variables, in this section, we will use the method of instrumental variables to overcome possible endogenous problems. We choose the fathers’ job position (fjob) as instrumental variables for the housing number variable. In the survey, the highest positions of parents’ jobs are divided into 10 categories. (1) Ordinary employees; (2) Leader in charge of a department; (3) Leader in charge of a unit; (4) (Deputy) Team Leader; (5) (Deputy) Section Director; (6) (Deputy) Director; (7) (Deputy) Director and above; (8) Village cadres; (9) Township cadres; (10) Farmers; (11) No job; (12) Others. We will take the answer of (1), (10), (11), and (12) (no job titles) as 0, and others are taken as 1. On the one hand, the job position of fathers is closely related to the number of housing properties of the respondents. If the father has a higher position, then their children can get more resources and have a higher probability to own more numbers of housing properties. On the other hand, the job positions of fathers do not link to the willingness to pay for green houses of their child, so we believe that these two instrumental variables are not related to the willingness of the respondents.

From Table 4, we can see that the job position of the father has a significant positive effect on the number of houses owned by the respondents.

**TABLE 4:** The ivprobit regression results of the first stage.

housenumber	Coefficient	SE	*t*	*P* > *t*	[95% confidence interval]	
fjob	0.09708	0.012337	7.87	0.000	0.072897	0.121263
lnhouseprice	0.042732	0.003332	12.82	0.000	0.0362	0.049264
gender	0.005296	0.009882	0.54	0.592	–0.01407	0.024666
education	1.20E−01	1.14E−02	10.51	0.000	9.73E−02	1.42E−01
marriage	–0.0117	0.026218	–0.45	0.656	–0.06309	0.039694
size	0.024961	0.003627	6.88	0.000	0.017851	0.032072
lnincome	0.007849	0.003335	2.35	0.019	0.001313	0.014386
rural	–0.00855	0.013492	–0.63	0.526	–0.03499	0.017898
_cons	0.468691	0.05834	8.03	0.000	0.354336	0.583047
*R*-square	0.0447					

The dependent variable is house number. z-Statistics are in parentheses, ***, **, and * indicate the significance levels of 1, 5, and 10%, respectively.

Table 5 reports the regression results of the second stage of ivprobit estimation (initial instrumental variable test). From Table 5, we can see the Wald test result of the exogenous null hypothesis “H0:ρ = 0,” the *P*-value is 0.0000, so house number can be considered as an endogenous explanatory variable at the 1% level. According to the estimation results in Table 3, the coefficient of the house number variable is 0.117, which is significant at the 1% level; but the ivprobit estimation result in Table 5 shows that the coefficient of the house number variable is 1.51, which is also significant at the 1% level. The above results show that if the general probit model is used for estimation, the endogenousness of house number will be ignored, which will underestimate the influence of the number of houses on risk attitudes.

**TABLE 5:** Regression results of the second stage of ivprobit estimation.

	Coefficient	SE	*z*	*P* > *z*	[95% confidence interval]	
housenumber	1.513887	0.347892	4.35	0.000	0.832031	2.195743
lnhouseprice	–0.00781	0.018158	–0.43	0.667	–0.0434	0.027778
gender	–0.03528	0.027316	–1.29	0.197	–0.08882	0.018258
education	1.84E−01	5.42E−02	3.4	0.001	7.82E−02	0.290523
marriage	–0.03346	0.073127	–0.46	0.647	–0.17679	0.109867
size	–0.04816	0.013206	–3.65	0.000	–0.07404	–0.02228
lnincome	–0.00407	0.009633	–0.42	0.673	–0.02294	0.014815
rural	–0.11323	0.037908	–2.99	0.003	–0.18753	–0.03893
_cons	–1.8436	0.228448	–8.07	0.000	–2.29135	–1.39586
Wald test of exogeneity	χ ^2^(1) = 22.53	*P* = 0.0000				

The dependent variable is willingness. z-Statistics are in parentheses, ***, **, and * indicate the significance levels of 1, 5, and 10%, respectively.

As can be seen from Table 6 the model setting passed a series of tests. First, regarding the unrecognizable test, the *P*-values of Kleibergen-Paap rk LM statistic is 0.000, rejecting the null hypothesis, indicating that there is a correlation between instrumental variables and endogenous variables. Second, regarding the test of weak instrumental variables, the *P*-values of CLR, KJ, AR, and Wald are all significant at the 1% level, so the null hypothesis “H0: Endogenous variables and instrumental variables are not correlated” should be rejected, and alternative hypotheses “H1: Endogenous variables are related to instrumental variables” should not be rejected. This also shows that the instrumental variables selected in this article are not weak instrumental variables.

**TABLE 6:** Ivprobit test.

	Test	Statistic	*P*-value
Unrecognizable test	Kleibergen-Paap rk LM	47.322	0.0000
Weak tool identification test	CLR	6.99	0.0089
	K	5.75	0.0165
	J	9.46	0.0021
	K-J	n.a.	0.0105
	AR	15.21	0.0005
	Wald	8.72	0.0031

## Mechanism Analysis

The above analysis finds that housing assets have indeed significantly affected the willingness of household heads to pay for green house. The greater the number of housing properties owned, the higher the willingness to pay for the green house of the respondents. Next, we will further verify whether the number of housing assets will affect the willingness of respondents to pay for the green house by the way of individual happiness.

From the perspective of the characteristics of the respondents, owning housing assets may affect the happiness of an individual, which in turn affects the willingness of the head to pay for a green house. Due to the housing wealth effect brought about by rising housing prices, people with more housing assets are more likely to have higher happiness brought by wealth, which may cause an influence on the purchase intention of respondents.

In the survey, there is a question that “in general, do you feel happy now?” We set the answer “very unhappy” is 1, “unhappy” is 2, “generally” is 3, “happy” is 4, and “very happy” is 5.

The above empirical results show that after adding the interaction term (hh) of the number of houses (house number) multiplied by happiness (happiness), the number of houses does not significantly affect the willingness of the respondents, but its interaction term significantly positively affects the willingness. That is one of the channels through which housing assets affect the willingness of householders is their happiness (see Table 7).

**TABLE 7:** Mechanism analyzing results.

	Variables	OLS	Logit	Probit
willingness	housenumber	0.0132	0.0522	0.032
		−0.974	−0.907	−0.899
	hh	0.00670**	0.0286**	0.0178**
		−2.165	−2.169	−2.179
	lnhouseprice	0.0143***	0.0636***	0.0384***
		−5.841	−5.879	−5.86
	gender	0.0008	0.00316	0.00183
		−0.111	−0.102	−0.0958
	education	0.127***	0.518***	0.324***
		−15.19	−14.79	−14.83
	marriage	−0.0677**	−0.315**	−0.189**
		(−2.454)	(−2.500)	(−2.500)
	size	0.00780***	0.0342***	0.0217***
		−3.157	−3.225	−3.309
	lnincome	0.00199	0.00842	0.00523
		−0.803	−0.795	−0.798
	rural	−0.0648***	−0.288***	−0.176***
		(−6.493)	(−6.551)	(−6.570)
		−2.382	(−9.173)	(−9.213)
	Constant	0.102**	−1.712***	−1.052***
	*R*-squared	0.033		

z-Statistics are in parentheses, ***, **, and * indicate the significance levels of 1, 5, and 10%, respectively.

## Robustness Test

This part conducts the robustness test by replacing the market house price with the average house price at the provincial level. It turns out that the number of housing assets still affect the willingness to pay for the green house of the respondents (see Table 8).

**TABLE 8:** Robustness test.

	Variables	OLS	Logit	Probit
willingness	housenumber	0.0419***	0.175***	0.108***
		−6.793	−6.652	−6.689
	lnhouseprice2	0.0429***	0.182***	0.112***
		−5.246	−5.221	−5.201
	gender	0.00172	0.00713	0.00423
		−0.248	−0.239	−0.23
	education	0.132***	0.543***	0.339***
		−16.56	−16.21	−16.22
	marriage	−0.0741***	−0.347***	−0.208***
		(−2.840)	(−2.881)	(−2.892)
	size	0.00962***	0.0419***	0.0264***
		−4.108	−4.161	−4.235
	lnincome	0.000929	0.00406	0.00244
		−0.389	−0.396	−0.385
	rural	−0.0855***	−0.381***	−0.232***
		(−9.699)	(−9.746)	(−9.763)
		(−1.372)	(−7.372)	(−7.370)
	Constant	−0.113	−2.581***	−1.599***
	*R*-squared	0.033		

z-Statistics are in parentheses, ***, **, and * indicate the significance levels of 1, 5, and 10%, respectively.

## Conclusion and Policy Implications

If a family has more housing assets, then the householder will have a higher willingness to buy a green house. Similarly, if the housing price is higher, householders are more willing to buy a green house.

In addition, in terms of the characteristics of the householder, the more educated the householder is, the stronger his willingness to buy a green house will be. Compared with unmarried people, married households are more likely to buy green houses. In addition, families in rural areas are more likely to buy green housings than those in urban areas.

### Mechanism Analysis Conclusion

The above analysis found that housing assets did significantly affect the willingness of householders to pay for green house. The more houses they own, the higher their willingness to pay for a green house will be. Then, we further verified whether the number of housing assets will affect the willingness of householders to pay for green housing through the way of individual happiness.

From the characteristics of householders, owning housing assets may affect individual happiness and then affect willingness of householders to pay for green houses. Due to the housing wealth effect brought by rising housing prices, people with more housing assets are more likely to have the happiness brought by higher wealth, which may affect the purchase intention of householders.

The above empirical results show that when the “housenumber” is multiplied by “hh” of “happiness,” the number of houses has no significant influence on the intention of householders, but the interaction has a significant positive influence on the intention. In other words, one of the channels through which housing assets affect the intentions of households is their happiness.

### Countermeasures and Suggestions at the Government Level

China is currently in the initial stage of green housing development. Mandatory policies are important ways to promote the improvement of green housing performance and marketing (Li et al., 2019). As the main body to promote the development of green housing, the government should play the main guiding and promoting role, that is, formulate strategic countermeasures from the macro level to promote the green housing development in a positive and healthy direction, and at the same time put forward some basic policies from the micro level, to stimulate willingness of consumers to buy green houses, and the development of the market demand side (Dong, 2013).

In the conclusion of this study, the higher the income and the more housing assets, the stronger the willingness to pay for consumers. Since the price of green housing is generally higher than that of normal houses, consumers will inevitably be restricted by their own consumption power when purchasing, and high-income groups are more able to afford higher-priced green houses. Therefore, specific incentive measures should be formulated for consumers to buy green houses, and practical preferences should be given to consumers of middle-and-low-income groups, for example, tax incentives, housing purchase subsidies, and property management incentives (Huang, 2014), that means providing consumers with economic stimulus from many aspects, and use the leverage principle of the market economy to motivate consumers to buy green houses.

At the same time, the research in this article found that people with higher education levels are more likely to buy green buildings. Generally, people with higher education have a stronger awareness of energy conservation and environmental protection, and their high willingness to pay for green houses may be more out of their own sense of social responsibility. This is consistent with the research conclusions of Kahn (2002). Kahn pointed out that the proportion of urban residents who went to college is significantly positively correlated with the local support for government environmental protection policies, because people with higher education are more aware of the long-term harm of environmental problems, and thus they pay more attention to environmental protection. Therefore, the government should use advantageous media platforms to strengthen the publicity and promotion of green housing, so as to create a green atmosphere in the whole society, such as introducing environmental friendliness, low lifetime cost, healthy and comfortable living characteristics of green housing from a technical point of view, so as to enhance consumers’ awareness of green houses, attract consumers’ attention, and arouse consumers’ expectation of buying green buildings. In short, it is to activate the demand side of the green housing market (Zhang et al., 2018).

In addition, we found that one of the channels through which the housing assets affect intentions of households is their happiness. Due to the continuous increase in Chinese housing prices in recent years, the wealth effect brought by housing assets has led to a sharp increase in the wealth of people who own more houses. In this way, the happiness index brought by wealth will also increase. Therefore, the government can introduce more policies to improve the happiness index of residents, including housing subsidies, affordable housing, and low-rent housing.

### Countermeasures and Suggestions at the Developer Level

As an important part of the residential market, developers play an important role in the promotion and development of green houses. Developers should abide by the rules and regulations of the government, follow the development trend of the residential market, and actively promote the development of green housing.

First, developers should respond to the policy of government, actively develop green housing, fulfill the government’s requirements for energy saving, water saving, and material saving for housing with high quality and quantity, seize the opportunity of the government to promote the development of green housing, and launch high-quality housing. Green residential real estate should seize the opportunity of green residential development, and expand their own influences (Li et al., 2019).

Second, the developer should cooperate with the government to actively create a green atmosphere for the whole society, vigorously promote environmental protection, and economic advantages of green housing. They can carry out special green housing real estate promotion activities, which can expand the brand influence of the developer, can improve its own visibility, and more importantly, can popularize the knowledge of green housing. Therefore, more consumers can understand green housing and the advantages of green housing, which will stimulate consumer expectations, strengthen consumer awareness, and increase consumer willingness to pay for green housing (Denni et al., 2018).

More importantly, many real estate brokers have noticed that modern buyers are becoming more and more selective, so before the implementation of advertising for green housing, the real estate company should not only consider individual differences of potential buyers, the desired features of the property, and how the location impacts on the property, but also pay more attention to the arousal and valence, affective behavior, emotional, and physiological states of possible buyers of green housing (AVABEPS) while they review the advertising. In other words, real estate advertising cannot ignore considering the physiological, emotional, and affective responses of clients, it is better to employ a neuro decision matrix, make multivariant planning performed on customized, video neuro-advertising green-housing variants, and multiple criteria analysis (Kaklauskas et al., 2020).

## Data Availability Statement

The original contributions presented in the study are included in the article/supplementary material, further inquiries can be directed to the corresponding author/s.

## Author Contributions

QW and ZZ: doing empirical analysis and writing the original draft together. WL: conceptualization and validation. All authors contributed to the article and approved the submitted version.

## Conflict of Interest

The authors declare that the research was conducted in the absence of any commercial or financial relationships that could be construed as a potential conflict of interest.

## Publisher’s Note

All claims expressed in this article are solely those of the authors and do not necessarily represent those of their affiliated organizations, or those of the publisher, the editors and the reviewers. Any product that may be evaluated in this article, or claim that may be made by its manufacturer, is not guaranteed or endorsed by the publisher.

## Appendix

Appendix (part of the questionnaire).

1. What is your gender?

   1. Male 2. Female

2. What is your birth year? _____
3. Which province are you from? _____
4. What is your educational level?

   1. No schooling at all 2. Primary school 3. Junior high 4. High school

   5. Technical high school 6. College/vocational school 7. Bachelor’s degree

   8. Master’s degree 9. Doctorate degree

5. What is your marital status at present?

   1. Unmarried 2. Married 3. Divorced 4. Remarried

6. Apart from yourself, how many family members live with you? _____
7. Are you living in rural or urban area?

   1. Rural 2. Urban

8. What is the type of your working role?

   1. An employee 2. An employer 3. An individual worker 4. A household worker

9. What was the highest position of your father?

   1. Ordinary staff/worker 2. Division leader of the work unit, e.g., manager

   3. Top leader of the work unit, e.g., general manager 4. (sub) Team Leader

   5. (deputy) Section chief 6. (deputy) Director of a division

   7. (deputy) Director-general of a bureau and above 8. Village cadre

   9. Township cadre 10. Peasant

   A. Others (please specify) B. He/she had no jobs before

10. What is the total income of your family last year? ______

   Or Which range below is the total income of your family last year approximately?

   1. Less than 10,000 2. 10,000–20,000 3. 20,000–50,000 4. 50,000–100,000

   5. 100,000–200,000 6. 200,000–300,000 7. 300,000–500,000 8. 500,000–1,000,000

   9. 1,000,000–2,000,000 10. 2,000,000–5,000,000 11. More than 5,000,000

11. How many houses do you own with rental houses excluded? (number of houses owned if you are the householder) _____
12. How much is this house worth currently (market price on real estate website)? (Unit: RMB) _____

   Or Which range below is the market price of house approximately?

   1. Below 10,000 2. 10,000–30,000 3. 30,000–50,000 4. 50,000–70,000 5. 70,000–100,000 6. 100,000–300,000 7. 300,000–500,000 8. 500,000–1,000,000 9. 1,000,000–5,000,000 10. 5,000,000–10,000,000 11. 10,000,000–15,000,000 12. 15,000,000–20,000,000 13. Above 20,000,000

13. Are you willing to pay for green housings? (China Green Building Evaluation Standard: land saving and outdoor environment, energy saving and energy utilization, water saving and water resource utilization, material saving and material resource utilization, indoor environmental quality, operation management, and construction management)

   1. Yes 2. No

14. In general, do you feel happy now?

   1. Very happy 2. Happy 3. Generally 4. Unhappy 5. Very unhappy

## References

[B1] Assessment Standard for Green Building (2016). *Assessment Standard for Green Building.* Beijing: MOHURD. Available online at: http://www.mohurd.gov.cn/wjfb/201601/t20160126_226447.html

[B2] Attaran S. Celik B. G. (2015). Students’ environmental responsibility and their willingness to pay for green buildings. *Int. J. Sustainabil. Higher Educat.* 2015 327–340. 10.1108/ijshe-04-2013-0029

[B3] Belaid F. Garcia T. (2016). Understanding the spectrum of residential energy-saving behaviours: French evidence using disaggregated data. *Energy Econom.* 57 204–214. 10.1016/j.eneco.2016.05.006

[B4] Buiter W. H. (2008). “Housing wealth isn’t wealth,” in *NBER Working Papers 14204*, (Cambridge, MA: National Bureau of Economic Research, Inc).

[B5] Carroll C. Slacalek J. Tokuoka K. White M. N. (2017). The distribution of wealth and the marginal propensity to consume. *Quantit. Econom.* 8 977–1020. 10.3982/qe694 30516143

[B6] Case K. E. Quigley J. M. Shiller R. J. (2005). Comparing Wealth Effects: The Stock Market versus the Housing Market. *Adv. Macroeconom.* 5 1–34.

[B7] Chen J. Hardin W. III Hu M. (2020). Housing, wealth, income and consumption: China and homeownership heterogeneity. *Real Estate Econom.* 48 373–405. 10.1111/1540-6229.12245

[B8] Chen X. R. Li R. Y. Wu X. (2021). Multi-homeownership and household portfolio choice in urban China. *J. Housing Built Environ.* 36 131–151.

[B9] CABEE (2017). *China building energy consumption research report.* Availbale online at: http://www.sohu.com/a/208615242_99960447

[B10] Data D. (2016). *Analytics and United Technologies. World Green Building Trends 2016.* Developing Markets Accelerate Global Green Growth.

[B11] Darko A. Chan A. P. C. (2016). Critical analysis of green building research trend in construction journals. *Habitat Int.* 57 53–63. 10.1016/j.habitatint.2016.07.001

[B12] Denni A. Lay P. Fandy T. Lin Y. (2018). Exploring consumers’ purchase intention towards green products in an emerging market: The role of consumers’ perceived readiness. *Int. J. Consumer Stud.* 42 389–401. 10.1111/ijcs.12432

[B13] Ding Z. H. Wang G. Q. Liu Z. H. Long R. Y. (2017). Research on differences in the factors influencing the energy-saving behavior of urban and rural residents in China–A case study of Jiangsu Province. *Energy Policy* 100 252–259. 10.1016/j.enpol.2016.10.013

[B14] Ding Z. Jiang X. Liu Z. Long R. Xu Z. Cao Q. (2018). Factors affecting low-carbon consumption behavior of urban residents: a comprehensive review. *Resour. Conserv. Recycl.* 132 3–15. 10.1016/j.resconrec.2018.01.013

[B15] Dong C. (2013). Discussion on the implementation of green housing problems and development countermeasures. *Building Economy* 1 87–90.

[B16] Fornara F. Pattitoni P. Mura M. Strazzera E. (2016). Predicting intention to improve household energy efficiency: The role of value-belief-norm theory, normative and informational influence, and specific attitude. *J. Environ. Psychol.* 45 1–10. 10.1016/j.jenvp.2015.11.001

[B17] Hu H. Geertman S. Hooimeijer P. (2014). The willingness to pay for green apartments: The case of Nanjing, China. *Urban Stud.* 51 3459–3478. 10.1177/0042098013516686

[B18] Hu H. Xu J. Zhang X. (2020). The role of housing wealth, financial wealth, and social welfare in elderly households’ consumption behaviors in China. *Cities* 96:102437. 10.1016/j.cities.2019.102437

[B19] Huang H. L. (2014). Empirical study on consumption choice of green housing. *Econom. Manag. Stud.* 12 58–65.

[B20] Jayantha W. M. Ming J. L. (2016). Buyers’ property asset purchase decisions: an empirical study on the high-end residential property market in Hong Kong. *Int. J. Strategic Property Manage.* 20 1–16. 10.3846/1648715x.2015.1105322

[B21] Jones S. A. Laquidara-Carr D. (2016). *World Green Building Trends 2016—Developing Markets Accelerate Global Green Growth.* Bedford (US): Dodge Data & Analytics.

[B22] Kahn M. E. (2002). Demographic change and the demand for environmental regulation. *J. Policy Anal. Manage.* 21 45–62. 10.1002/pam.1039

[B23] Kaklauskas A. Lepkova N. Raslanas S. Vetloviene I. Milevicius V. Sepliakov J. (2021). COVID-19 and green housing: a review of relevant literature. *Energies* 14 1–41. 10.3390/en14082072

[B24] Kaklauskas A. Zavadskas E. K. Schuller B. Lepkova N. Dzemyda G. Sliogeriene J. (2020). Customized ViNeRS method for video neuro-advertising of green housing. *Int. J. Environ. Res. Public Health* 17 1–28. 10.3390/ijerph17072244 32230704 PMC7177231

[B25] Li J. (2016). Energy performance heterogeneity in China’s buildings sector: a data-driven investigation. *Renew. Sustain. Energy Rev.* 58 1587–1600. 10.1016/J.RSER.2015.12.326

[B26] Li Q. W. Ruyin L. Hong C. (2018). Differences and influencing factors for Chinese urban resident willingness to pay for green housings: Evidence from five first-tier cities in China. *Appl. Energy* 229 299–313. 10.1016/j.apenergy.2018.07.118

[B27] Li Q. Li W. Long R. Chen H. Chen F. Cheng X. (2019). Chinese urban resident willingness to pay for green housing based on double-entry mental accounting theory. *Natural Hazards* 95:4.

[B28] Liao W. C. Zhao D. Sing T. F. (2014). Risk attitude and housing wealth effect. *J. Real Estate Finance Econom.* 48 467–491. 10.1007/s11146-013-9407-2

[B29] Lindén A. L. Annika C. Bjorn E. (2006). Efficient and inefficient aspects of residential energy behaviour: What are the policy instruments for change? *Energy Policy* 34 1918–1927. 10.1016/j.enpol.2005.01.015

[B30] Ma M. Yan R. Cai W. (2017). A STIRPAT model-based methodology for calculating energy savings in China’s existing civil buildings from 2001 to 2015. *Nat. Hazards* 87 1–17. 10.1007/s11069-017-2847-x

[B31] Peng C. Qiu W. Song Q. Huang B. (2019). Housing wealth appreciation and consumption: evidence from China. *J. Asia Pacific Economy* 24 556–577. 10.1080/13547860.2019.1661570

[B32] Pratt J. W. Zeckhauser R. J. (1987). Proper risk aversion. *Econometr. J. Econometr. Soc.* 1987 143–154. 10.2307/1911160

[B33] Robinson S. Simons R. Lee E. Kern A. (2016). Demand for Green Buildings: Office Tenants’ Stated Willingness-to-Pay for Green Features. *J. Real Estate Res.* 38 423–452. 10.1080/10835547.2016.12091450

[B34] Rosner Y. Zohara A. Amotz P. (2021). Consumer’s attitude, socio-demographic variables and willingness to purchase green housing in Israel. *Environ. Dev. Sustainabil.* 2021:8.

[B35] Sinai T. Souleles N. S. (2005). Owner-occupied housing as a hedge against rent risk. *Quart. J. Econom.* 120 763–789. 10.1093/qje/120.2.763

[B36] Wang K. Yu S. Li M. Wei Y.-M. (2015). Multi-directional efficiency analysis-based regional industrial environmental performance evaluation of China. *Nat. Hazards* 75 273–299. 10.1007/s11069-014-1097-4

[B37] Wu L. Y. (2000). Architecture in the new millennium. *J. Architect.* 5 9–19. 10.1080/136023600373655

[B38] Yang J. Li H. (2014). Research on demand Factor System of green building based on consumers’ willingness to pay. *Build. Economy* 10 104–108.

[B39] Zhang L. Cai S. (2015). Who is more willing to buy green House: The effect of resident characteristics on willingness to pay for green House. *China Real Estate* 18 23–31.

[B40] Zhang L. Chen L. W. Wu Z. Z. Xue H. Dong W. (2018). Key Factors Affecting Informed Consumers’ Willingness to Pay for Green Housing: A Case Study of Jinan, China. *Sustainability* 10:1711. 10.3390/su10061711

[B41] Zhang R. H. (2011). *Research on affordability of Green Building.* Harbin: Harbin Institute of Technology.

[B42] Zhao S. W. Chen L. W. (2020). Influencing factors and mechanism of green housing purchase intention-Based on grounded Theory. *Enterpr. Economy* 4 28–36. 10.1016/j.indic.2021.100164

[B43] Zhao S. W. Chen L. W. (2021). Exploring Residents’ Purchase Intention of Green Housings in China: An Extended Perspective of Perceived Value. *Int. J. Environ. Res. Public Health* 18:4074. 10.3390/ijerph18084074 33924311 PMC8069697

